# T-Cell Proapoptotic and Antifibrotic Activity Against Autologous Skin Fibroblasts *in vitro* Is Associated With IL-17A Axis Upregulation in Systemic Sclerosis

**DOI:** 10.3389/fimmu.2020.00220

**Published:** 2020-02-27

**Authors:** Serena Vettori, Giusi Barra, Barbara Russo, Alessia Borgia, Giuseppe Pasquale, Luciana Pellecchia, Lucia Vicedomini, Raffaele De Palma

**Affiliations:** ^1^Rheumatology Unit, Department of Precision Medicine, University of Campania “Luigi Vanvitelli”, Naples, Italy; ^2^Clinical Immunology Unit, Department of Precision Medicine, University of Campania “Luigi Vanvitelli”, Naples, Italy; ^3^Institute of Protein Biochemistry (IBP-CNR), Naples, Italy

**Keywords:** systemic sclerosis, T cells, fibroblasts, co-cultures, apoptosis, IL-17A, IL-17RA, chemokines

## Abstract

**Background:** Systemic sclerosis (SSc) T cells can induce apoptosis of autologous skin fibroblasts *in vitro*. Th17 cells have been reported to increase in SSc patients, and interleukin-17A (IL-17A) has a profibrotic function. We used a system based on T-cell-autologous fibroblast co-cultures to further investigate a possible role of IL-17A in SSc.

**Methods:** T cells from diffuse SSc patients were co-cultured with autologous skin fibroblasts. *IL17A* mRNA was assessed by real-time PCR in co-cultured and control T cells, while *IL17RA, CXCL1, CCL2, CCL3, COL1A1, COL3A1, CTGF, TGFBR2*, and *SMAD3* mRNAs were assessed in co-cultured and control fibroblasts. In subset experiments, co-cultures and control cells were treated with either IL-17A or IL-17A *plus* anti-IL17 receptor monoclonal antibody (α-IL-17RA mAb). Chemokine and procollagen type I (PCI) production was further investigated at the protein level in cell culture supernatants by multiple suspension immunoassay and sandwich ELISA, respectively. Co-cultured and control fibroblasts were also stained with Annexin V and analyzed by flow cytometry.

**Results:** T cell–fibroblast co-cultures overexpressed *IL17A* and *IL17RA*. Furthermore, co-cultured fibroblasts upregulated IL-17A targets *CXCL1, CCL2*, and *CCL3*, while *COL1A1, COL3A1, CTGF*, and two key effectors of the TGF-β signaling, *TGFBR2* and *SMAD3*, were found downregulated. Consistently, chemokine concentrations were increased in co-culture supernatants, while PCI levels were reduced, especially after stimulation with ectopic IL-17A. Finally, simultaneous α-IL-17RA mAb treatment restored PCI levels and reduced fibroblast apoptosis in IL-17A-stimulated co-cultures.

**Conclusion:** These data suggest that IL-17A upregulation might play a role in modulating T cell-mediated antifibrotic and proapoptotic effects in co-cultured autologous skin fibroblasts.

## Introduction

Systemic sclerosis (SSc) is a chronic multiorgan disease characterized by microvascular injury, autoimmune phenomena, and fibroblast activation, leading to uncontrolled extracellular matrix (ECM) deposition in the skin and visceral organs and increased mortality due to vital organ dysfunction (1, 2). The etiopathogenesis of the disease remains unknown. However, endothelial, immune cell, and fibroblast activation, and most notably T cell–fibroblast cross-talk, play a role (3). In the past years, we and others showed that the skin of patients with diffuse SSc (dcSSc) of recent onset (<3 years) is predominantly infiltrated by oligoclonally expanded CD4+ T cells (4, 5). Furthermore, we demonstrated that SSc T cells co-cultured with autologous skin fibroblasts up to 10 days display the same clonotypes found in the skin of these patients and can induce fibroblast apoptosis *in vitro* (6). These data suggest that an antigen-driven T-cell response could be initially devoted to control the aberrant fibroblast activation found in SSc. In line with our observations, other authors reported that chemically pre-activated peripheral γδT cells from SSc patients can also induce autologous fibroblast apoptosis after a short-term exposure *in vitro* (7). In those experiments, both T cell–fibroblast interaction and activation of γδT cells was paralleled by a cytokine burst in which profibrotic cues were more prominent (5, 6, 8, 9). These events may account for the escape of fibroblasts from a control attempted by T cells and for the resistance to apoptosis *in vivo*.

More recent evidences suggest that, among other immune-inflammatory mediators, interleukin-17A (IL-17A) and Th17 could play a key function in the pathogenesis of SSc (10), even though their role remains unclear (11). IL-17A seems to promote the production of proinflammatory mediators and metalloproteinases in human fibroblasts. Moreover, this cytokine suppresses collagen type I and connective tissue growth factor (CTGF) synthesis and counteracts alpha-smooth muscle actinin (α-SMA)-mediated myofibroblast transdifferentiation *in vitro* (12–15). However, in animal models of lung and skin fibrosis, IL-17A deficiency seems to protect against collagen deposition (16–18), and IL-17A stimulation of mouse fibroblast lines induces CTGF and transforming growth factor-β (TGF-β) overexpression (18). A possible explanation for this dual role of IL-17A in SSc has been provided by Dufour et al., who showed that IL-17A plays different effects on cultured SSc fibroblasts, depending on the prevalent activation of either IL-17A or TGF-β signaling pathway (19). In this context, the assessment of IL-17A levels in our co-cultures and a better knowledge of the effects of its modulation in T cell–fibroblast co-cultures could be helpful to add details on T cell–fibroblast dynamics in SSc *in vitro*.

## Method

### Patients

Nine dcSSc patients (20) with a disease duration from Raynaud's phenomenon (RP) onset ≤ 3 years, all meeting ACR/EULAR 2013 classification criteria (21), underwent whole blood drawing for peripheral blood mononuclear cell (PBMC) isolation and punch skin biopsy as described elsewhere according to international standards (6, 22), after giving informed written consent. Supplementary Table 1 summarizes demographic, clinical, and serological features of the patients enrolled. All biospecimens were obtained at first observation before initiating any immunomodulatory treatment.

The study protocol was approved by the Ethics Committee of the Department of Internal and Experimental Medicine “F.Magrassi-A. Lanzara” of the Second University of Naples (protocol n. 303/March 16th 2017).

### Cultures and Reagents

Fibroblasts were obtained by outgrowth culture from two to three 1-mm^2^ skin pieces into Petri dishes with DMEM added with 10% heat-inactivated fetal calf serum (FCS), 1 mM penicillin–streptomycin, and 2 mM l-glutamine. After 2–3 weeks, cells reached confluence, were harvested by trypsin treatment, and re-seeded in RPMI1640. Cells at passages 3–7 were used to set co-cultures. For each experiment, at least five cell batches were assayed in duplicate. All reagents were purchased from Invitrogen, ThermoFisher Scientific Inc., Waltham, MA, USA.

PBMCs were isolated from 20 ml of peripheral blood by centrifugation in a density gradient (Ficoll-Hypaque, Pharmacia LKB Biotechnology, New Brunswick, NJ, USA), washed, counted, and co-cultured up to 10 days with autologous fibroblasts in a 10:1 ratio in the presence of human recombinant IL-2 (hrIL-2) at a concentration of 20 U/ml, as described previously (5, 6), to sustain fibroblast-induced T-cell expansion (23). Fibroblasts alone and PBMCs alone *plus* hrIL-2 were cultured separately as controls. In subset experiments, co-cultures were added with human recombinant IL-17A (hrIL-17A) at a concentration of 6.25 ng/ml and/or IL-17RA neutralizing monoclonal antibody (α-IL-17RA mAb) at a concentration of 16 μg/ml. Human BSA 0.1% and goat isotype IgG were used as irrelevant controls for IL-17A and α-IL-17RA mAb treatment, respectively (IL-17A from Gibco, ThermoFisher Inc., Waltham, MA, USA; α-IL-17RA from R&D Systems Inc., Minneapolis, MN, USA). Minimal effective concentrations of both hrIL-17A and α-IL-17RA mAb were assessed by titration experiments measuring target gene expression induction, according to manufacturer instructions (Supplementary Figure 1).

### RNA Isolation and Gene Expression Analysis

RNA isolation was performed on cultured cells after trypsinization, washing in PBS and addition of Trizol Reagent (Invitrogen, ThermoFisher Scientific Inc., Waltham, MA, USA) according to manufacturer instructions. Then, 100 ng of total RNA was reverse transcribed using random hexamers, Multiscribe reverse transcriptase 50 U/μl, RT buffer, dNTPs, and RNase inhibitor 20 U/μl. 18S rRNA was used as endogenous control and analyzed by TaqMan-based real-time PCR using a pre-developed FAM-labeled primer-probe system (Hs99999901_s1; Applied Biosystems, ThermoFisher Scientific Inc., Waltham, MA, USA), while *IL17A, IL17RA, IL17RC, CXCL1, CCL2, CCL3, TGFBR2, SMAD3, CTGF, COL1A1*, and *COL3A1* mRNAs were analyzed by real-time PCR in a Sybr Green Master Mix, using 1 μl of cDNA and 11.25 μM self-designed primer pairs (all Applied Biosystems, ThermoFisher Scientific Inc., Waltham, MA, USA). Samples without the enzyme in the RT reaction were used as negative controls to exclude genomic contamination, and the quality of the primer amplification was tested by dissociation curve analysis (24). For relative quantification, the comparative threshold cycle (Ct) method was used (25). Primer sequences are reported in Supplementary Table 2.

### Protein Analysis

CXCL1, CCL2, and CCL3 levels were measured in culture supernatants by immunosuspension assay. Tests were performed with spectrally encoded beads coupled with capture antibodies each specific to the analyte of interest as the solid support, and a biotinylated detection antibody–streptavidin–phycoerythrin complex, as the reporter system (Merk Millipore, Billerica, MA, USA) to be read by a double laser-based fluorimetric instrument (Luminex 200, Luminex Corporation, Austin, TX, USA). Concentrations obtained by interpolation of sample fluorescence intensities with a standard curve were expressed as pg/ml.

Serum procollagen type I (PCI) levels were measured by sandwich ELISA and concentrations were also expressed in pg/ml by interpolation of sample optical densities with standard curves (Elabscience Biotechnology Co, Whu Han, PRC).

### Apoptosis Analysis

The Annexin V-FITC-labeled Apoptosis Detection Kit (Roche Diagnostics GmbH, Penzberg, Germany) was used to detect and quantify apoptosis by flow cytometry according to the manufacturer instructions. Cells were collected by centrifugation for 10 min at 500 *g* and then re-suspended at a density of 1 × 10^5^ cells/ml in 1 binding buffer (HEPES buffer, 10 mM, pH 7.4, 150 mM NaCl, 5 mM KCl, 1 mM MgCl^2^, and 1.8 mM CaCl_2_) and stained simultaneously with FITC-labeled Annexin V and propidium iodide (PI). PI was used as a cell viability marker. Cells were analyzed using a FACScalibur flow cytometer.

### Statistical Analysis

GraphPad Prism software version 6.0 (GraphPad software Inc., San Diego, CA, USA) was used for statistical analysis. Continuous variables were expressed as mean ± SD and medians with interquartile range (IQR). The D'Agostino-Pearson test was applied to test normality data distribution and the Student's paired or unpaired *t*-test or the Mann–Whitney and the Wilcoxon tests were used as appropriate. The one-way ANOVA test was used for multiple group comparison. Results were considered statistically significant for *p* < 0.05.

## Results

### IL-17A Is Upregulated in Co-cultured PBMCs and IL-17RA Is Upregulated and Activated in Autologous Co-cultured Skin Fibroblasts From Early dcSSc Patients

**Figure 1:**
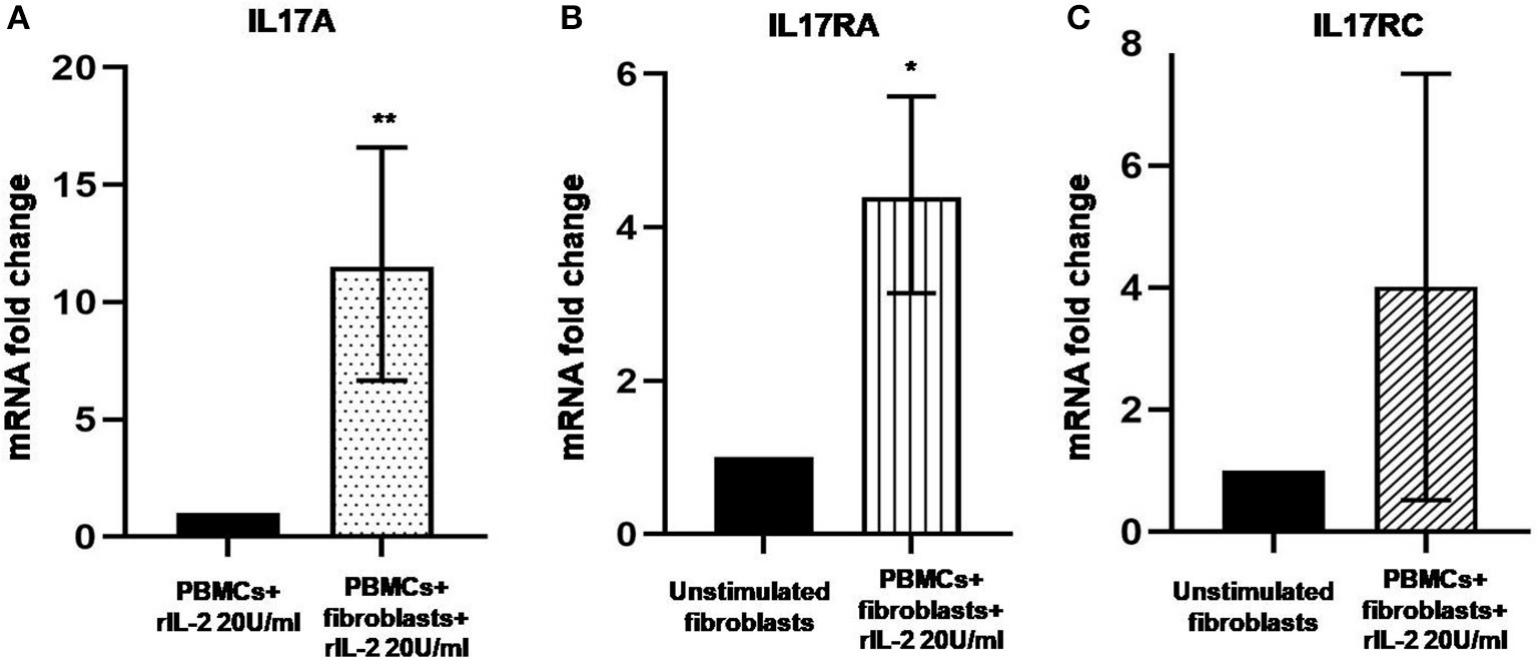
Levels of *IL17A, IL17RA*, and *IL17RC* in co-cultured PBMCs and fibroblasts from early dcSSc patients. Results are fold changes in mRNA levels of target genes in real-time PCR. **(A)** Shows the expression of *IL17A* in PBMCs co-cultured with autologous skin fibroblasts and hrIL-2 20 U/ml as compared to PBMCs cultured in the presence of hrIL-2 20 U/ml only. **(B)** Shows the expression of *IL-17RA* in fibroblasts co-cultured with autologous PBMCs and hrIL-2 20 U/ml as compared to fibroblasts alone. **(C)** Shows the expression of *IL-17RC* in fibroblasts co-cultured with autologous PBMCs and hrIL-2 20 U/ml as compared to fibroblasts alone. Data are median and IQR ranges, Wilcoxon signed rank test in panels **(A,B)**. Data are mean and SD, one sample *t*-test in panel **(C)** because of normal data distribution. *p < 0.05; **p < 0.01; number of samples per group = 6.

**Figure 2:**
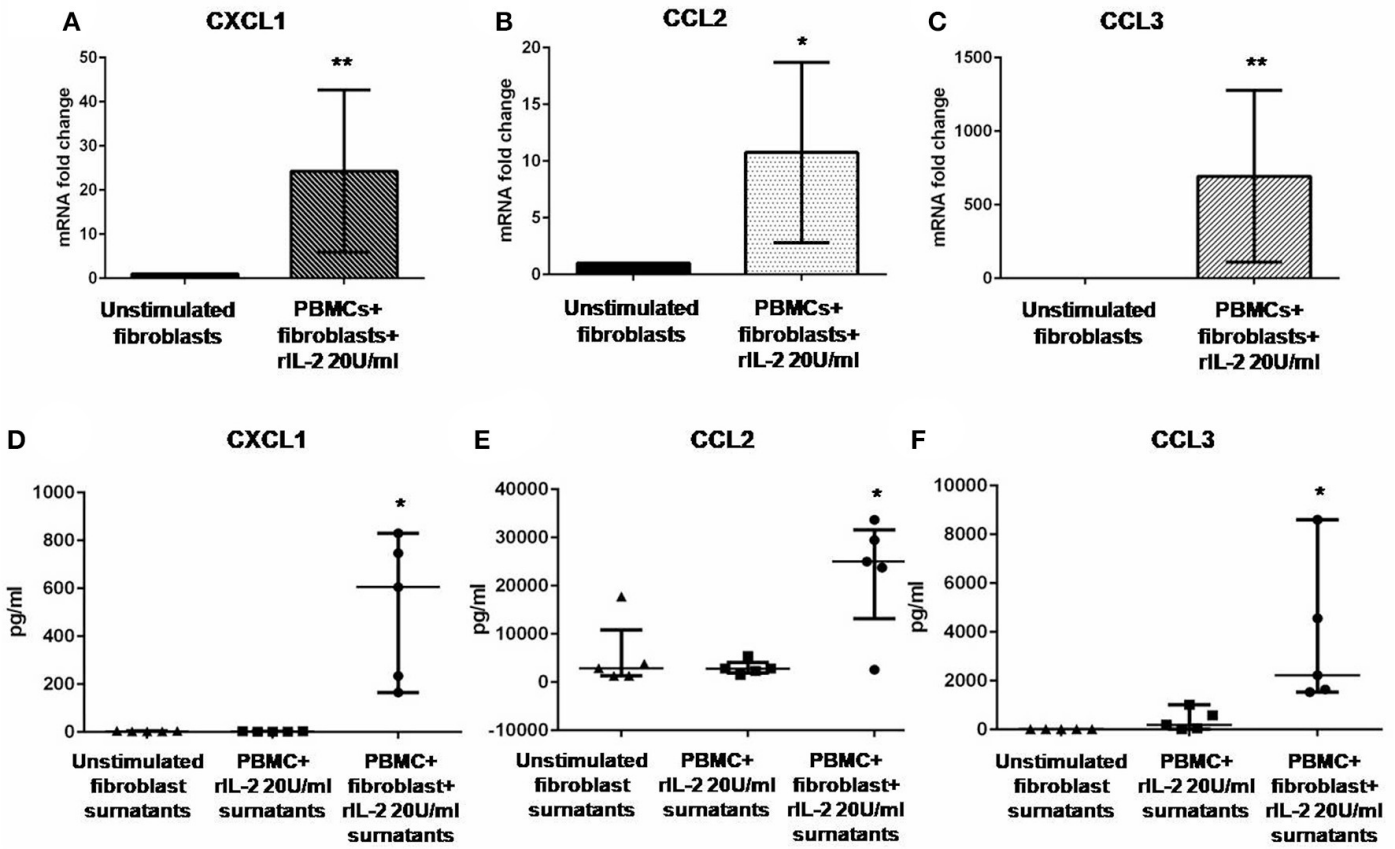
Levels of CXCL1, CCL2, and CCL3 in co-cultured fibroblasts and supernatants from early dcSSc patients. Results in panels **(A–C)** are fold changes in mRNA levels of IL17A target genes in real-time PCR. Results in panels **(D–F)** are concentrations expressed in pg/ml, in multiple suspension immunoassay. **(A–C)** Show the expression of *CXCL1, CCL2*, and *CCL3* in fibroblasts co-cultured with autologous PBMCs and hrIL-2 20 U/ml as compared to control fibroblasts. **(D–F)** Show the concentrations of secreted CXCL1, CCL2, and CCL3 in supernatants from co-cocultures and hrIL-2 20 U/ml as compared to supernatants from control fibroblasts after adjusting by concentrations measured in PBMCs cultured with hrIL-2 20 U/ml only. Data are median and IQR ranges, Wilcoxon signed rank test. *p < 0.05; **p < 0.01; number of samples per group = 5.

In Figure 1A, we show IL-17A expression in SSc PBMCs co-cultured with autologous skin fibroblasts. In these experiments, we found an increase in IL-17A mRNA levels by median 11.5-fold as compared to PBMCs cultured alone in the presence of hrIL-2 (*p* < 0.01), thus suggesting that IL-17A is secreted during T cell–fibroblast interactions in early dcSSc. This finding was consistent with the increased expression in the mRNA levels of both the subunit A of IL-17A receptor, IL-17RA, and the subunit C, IL-17RC, in the co-cultured fibroblasts by median 4.3-fold (*p* < 0.05) (Figure 1B), and mean 4-fold (Figure 1C), respectively, as compared to fibroblasts cultured alone. However, to investigate whether IL-17 receptor overexpression in co-cultured fibroblasts ensues in the activation of the receptor, a heteromeric structure made of at least one subunit IL-17RA that partners IL-17RC in IL-17A-mediated responses in autoimmune disorders (26), we investigated mRNA levels of several IL-17A target genes in the co-cultured fibroblasts, namely, *CXCL1, CCL2*, and *CCL3*, that were found increased by 29-fold, 11.9-fold, and 773.3-fold, respectively (*p* < 0.05) (Figures 2A–C). Consistently, the corresponding secreted proteins were increased by 11.2-fold, 8.9-fold, and 252.4-fold, respectively (*p* < 0.05), in supernatants from co-cultures as compared to supernatants from unstimulated fibroblasts (Figures 2D–F).

### IL-17RA Upregulation and Activation in Skin Fibroblasts Co-cultured With Autologous PBMCs Are Associated With the Downregulation of Pro-Fibrotic Genes in Early dcSSc

**Figure 3:**
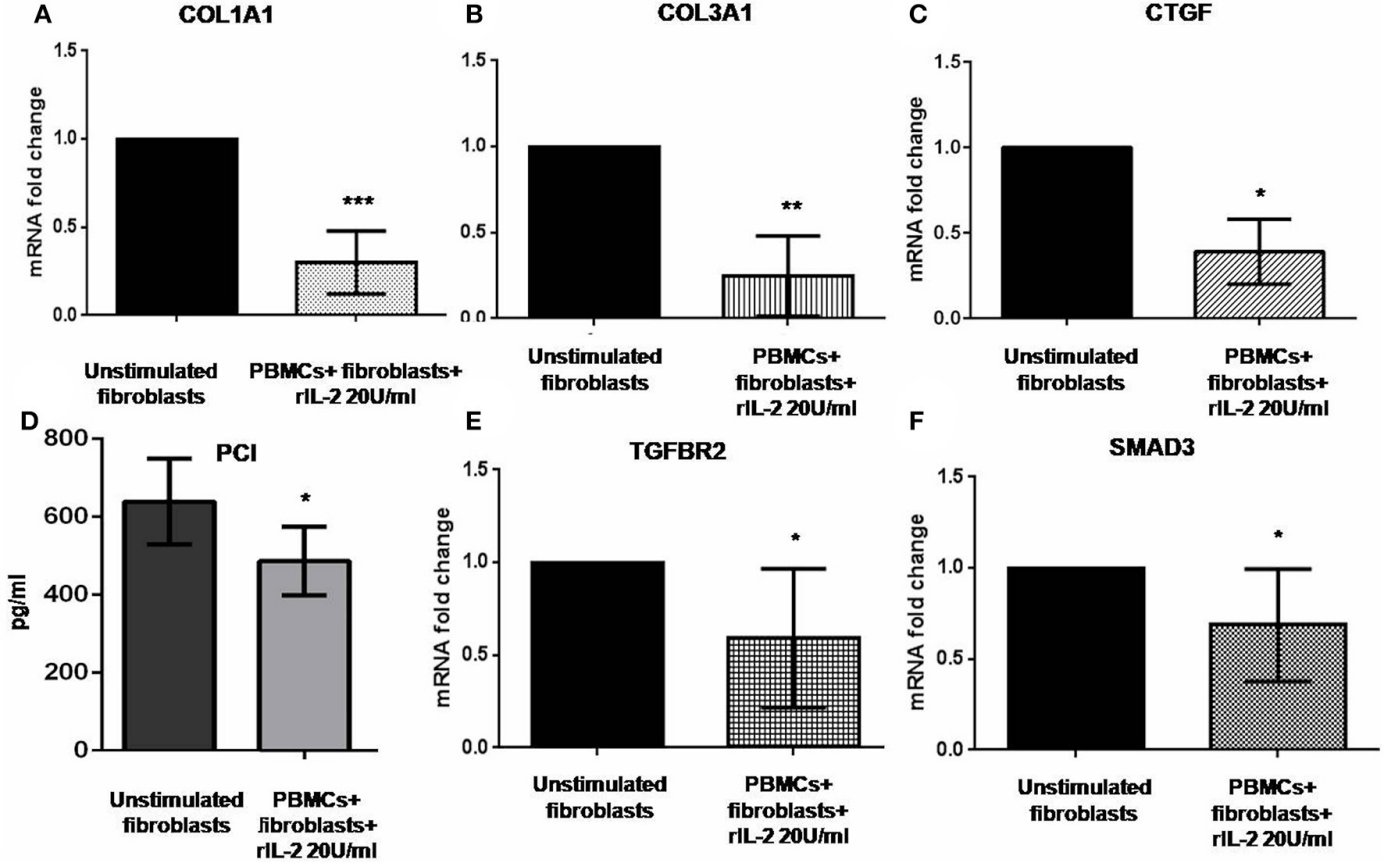
Profibrotic gene levels in co-cultured fibroblasts from early dcSSc patients and PCI in co-culture supernatants. Results in panels **(A–C)** and **(E,F)** are fold changes in mRNA levels of the profibrotic genes *COL1A1, COL3A1*, and *CTGF*, and of the two key mediators of the TGF-β signaling *TGFBR2* and *SMAD3* in real-time PCR. All show the expression of the investigated genes in fibroblasts co-cultured with autologous PBMCs and hrIL-2 20 U/ml as compared to control fibroblasts. Data are median and IQR ranges, Wilcoxon signed rank test. Results in panel **(D)** are concentrations as expressed in pg/ml in sandwich ELISA. Data are mean ± SD, paired Student's *t*-test. *p < 0.05; **p < 0.01; ***p < 0.001; number of samples per group = 5.

Previous reports suggested that IL-17A could negatively regulate type I collagen expression in humans (11, 27, 28) and we found that IL-17A was upregulated in our co-cultured PBMCs. Therefore, we looked at *COL1A1* and other profibrotic genes, namely, *COL3A1* and *CTGF* in SSc fibroblasts co-cultured with the autologous PBMCs overexpressing IL-17A. We found that all these genes were significantly downregulated in co-cultured fibroblasts as compared to control cells to 0.33-fold (*p* < 0.001), to 0.24-fold (*p* < 0.01), and to 0.31-fold (*p* < 0.05) (Figures 3A–C), respectively. Furthermore, the expression of two key molecules crucial to the TGF-β signaling pathway, *TGFBR2* and *SMAD3*, was downregulated as well to 0.59-fold and 0.79-fold (*p* < 0.05), respectively (Figures 3E,F). Finally, since type I collagen is the most abundant collagen type that accumulates in human skin in SSc (29), we measured concentration of PCI in supernatants from co-cultures as compared to supernatants from unstimulated fibroblasts. Here, we found a decrease of PCI levels in co-cultures from mean 639 ± 109.7 to 486.4 ± 88.16 pg/ml (*p* < 0.05) (Figure 3D), thus further confirming a functional effect due to IL-17A.

### Hampering IL-17A Signaling in Fibroblasts Co-cultured With Autologous PBMCs Affects Procollagen Type I Secretion and Protects Fibroblasts From Apoptosis in Early dcSSc

**Figure 4:**
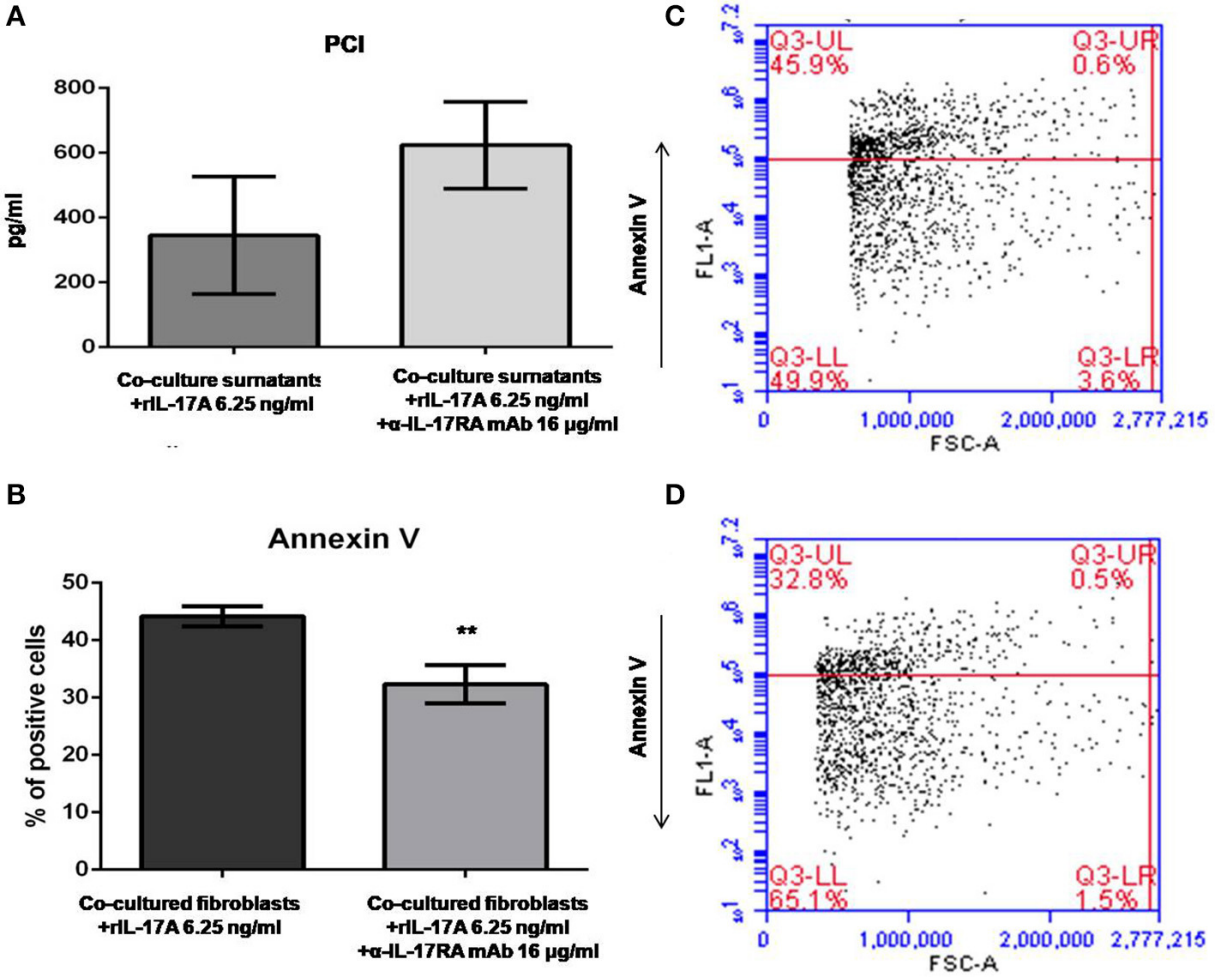
PCI concentration and SSc fibroblast apoptosis in co-cultures exposed to either IL-17A or IL-17A+α-IL-17A mAb. **(A)** Shows that concentrations of PCI in supernatants from SSc PBMC–fibroblast co-cultures treated with ectopic rhIL-17A 6.25 ng/ml are decreased, while simultaneous treatment of these co-cultures with ectopic rhIL-17A and a neutralizing mAb against IL-17RA subunit, α-IL-17A mAb16 μg/ml, increases PCI production (number of samples per group = 3). **(B)** Shows that α-IL-17A mAb at the same concentration is able to reduce significantly the apoptotic rate of co-cultured SSc fibroblasts exposed to rhIL-17A (number of samples per group = 5). Data are mean ± SD, paired Student's *t*-test. **p < 0.01. The scatterplot graph in panel **(C)** shows the percentage of apoptotic fibroblasts co-cultured with autologous PBMCs obtained from a representative SSc patient, added with ectopic rhIL-17A 6.25 ng/ml. The scatterplot graph in panel **(D)** shows the percentage of apoptotic fibroblasts co-cultured with autologous PBMCs obtained from the same representative SSc patient, added with both ectopic rhIL-17A 6.25 ng/ml and α-IL-17A mAb16 μg/ml.

Data generated so far prompted us to evaluate if the activation of IL-17A signaling pathway could be implicated in the *in vitro* dynamics between SSc PBMCs and autologous skin fibroblasts from early dcSSc patients. Different T-cell subsets can be present in these co-cultures playing complex interactions with each other and with fibroblasts. Therefore, to link PBMC antifibrotic activity and previously reported proapoptotic effects on co-cultured autologous fibroblasts, we looked at both PCI production and fibroblast apoptosis after stimulation of co-cultured cells, adding a suboptimal dose of ectopic hrIL-17A in order to maximize effects due to this cytokine, given the multiple components that may drive PBMC-induced apoptosis in co-cultured autologous fibroblasts. As further control, in a subset of experiments, we simultaneously administered α-IL-17RA mAb to co-cultures to counteract IL-17A signaling. In these experimental settings, co-cultured fibroblasts showed a reduced PCI production in the presence of hrIL-17A to levels lower than those measured in the presence of PBMCs and BSA 0.1% (354.2 ± 181.2 vs. 486.4 ± 88.16 pg/ml), even though the difference was not statistically significant. In addition, combined treatment with IL-17A and α-IL-17RA mAb allowed the measurement of PCI levels in co-culture supernatants similar to those found in supernatants from fibroblasts cultured alone (623.4 ± 133.6 pg/ml), although the difference with PCI levels from co-cultures with rhIL-17A and isotype control IgG was not statistically significant (Figure 4A). Most intriguingly, when we looked at co-cultured fibroblasts stained with annexin V and PI, we found that cells treated with rhIL-17A had an significantly increased expression of Annexin V, that was reduced from mean 44.2 to 32.3% (*p* < 0.01) in presence of α-IL-17RA mAb (Figure 4B). Figures 4C,D are scatterplot graphs from a representative SSc fibroblast-autologous PBMC co-culture showing the percentage of apoptotic fibroblasts (Annexin V positive) in presence of either rhIL-17A or rhIL-17A *plus* α-IL-17A, respectilvely.

## Discussion

We have previously reported that T cells of early SSc patients kill autologous fibroblasts *in vitro*, while we did not observe this phenomenon in the same experimental setting when we used cells from patients affected by different autoimmune diseases, such as systemic lupus erythematosus (5, 6). These data support the hypothesis that T cells undergo an antigen-driven oligoclonal expansion in the skin of SSc patients in the early phases of the disease toward the aberrantly activated fibroblasts (6, 7). Moreover, it is well-known that a T-cell response in SSc tissues is detectable only in the early disease stage, while an extensive fibrosis with a neglectable immune infiltrate is observed in late stages. Factors that drive the failure of such immune response *in vivo* are not known (3). Due to these observations, we wanted to further investigate the dynamics and the function of T cell–fibroblast interaction in SSc, using our experimental co-culture setting that we previously detailed (5, 6).

IL-17 has been proposed to have a central role in SSc (reviewed in 11). Analysis of our *in vitro* data demonstrated an increased production of IL-17A when T cells and autologous SSc skin fibroblasts were co-cultured. Starting from this finding, we moved to show a role of IL-17A, and its signaling pathway, in profibrotic gene modulation and in proapoptotic effects played by PBMCs co-cultured with autologous skin fibroblasts from early dcSSc patients.

Indeed, we found a downregulation of the mRNA levels of TGFBR2 and SMAD3 in co-cultured fibroblasts that could be, at least in part, associated with modulatory effects exerted by IL-17A on the TGF-β signaling, as recently described in other fibrotic disorders (30). A functional shift of the Th response toward a Th17 profile in SSc patients has been described by us and others (31, 32). Therefore, one possible explanation could be that skin microenvironment in SSc drives a Th17 polarization (10), thus promoting a Th polarization that, in the long term, is not able to eliminate altered SSc fibroblasts but concurs to the fibrosis development through the production of IL-17 (33, 34).

*In vitro*, data from other groups showed that, in human cells, IL-17A is able to induce a proinflammatory profile (12, 13, 35), including increased expression of CCL2 and CXCL8 chemokines (13, 35), interleukins IL-1β and IL-6 (12, 35), adhesion molecules like ICAM-1 and VCAM-1 (12, 35), and metalloproteinases like MMP-1 (14). Actually, all these factors have been implicated in the pathogenesis of SSc, even though their role seems to be strictly dependent on the phase of the disease (3, 11, 36). Moreover, recent data also indicate that, despite the fact that IL-17A shares high homology with IL-17F and is coordinately expressed, the effects in SSc skin and fibroblasts are restricted to the IL-17A isoform (37).

In a previous paper, we reported that in PBMC–fibroblast co-culture supernatants from early dcSSc patients, TGF-β levels are stably increased over the entire culture period (10 days) (6). In these new sets of experiments, we confirm the stable increase of *TGFB1* and also of *IL4* and *IL1B* mRNAs in PBMCs over the 10 days of co-culture (Supplementary Figure 2). This aspect partially explains the impairment of the immune response.

The potential antifibrotic activity of activated T cells has long been known (38, 39). This is the first report that describes such effects in a dynamic system like PBMC–fibroblast co-cultures, highlighting the role of IL-17A. Of note, in previous reports, activated T cell-derived particles specifically induced type I collagen inhibition (40) and, interestingly, also more recent data show that IL-17A antifibrotic effects seem to affect primarily type I collagen (13, 19, 27, 28). In our experiments, actually, we could demonstrate a downregulation also of the α1 chain of type III collagen at the mRNA level that should be further investigated.

As regards *CTGF* downregulation, data are consistent with the impaired expression of genes encoding for the TGF-β signaling components *TGFBR2* and *SMAD3*, as CTGF is one of the major downstream mediators of TGF-β (41). Consistently with our findings, Nakashima et al. reported that IL-17A is able to induce *COL1A1* and *CTGF* downregulation indirectly *via* the overexpression of miR-129-5p (15). Moreover, Truchetet et al. recently showed that IL-17A is able to prevent TGF-β-dependent myofibroblast differentiation *in vitro* (14).

It has to be underlined, however, that the hypothesized antifibrotic role of IL-17A in SSc is not supported by experimental models of inflammatory/drug-induced fibrosis, in which IL-17A seems to induce fibrosis in both skin and lung disease (16, 17, 42, 43). These discrepancies might depend on the fact that none of the mouse models that have been used to investigate the role of IL-17A in skin and lung fibrosis *in vivo* recapitulate the complete features of SSc. In particular, these animals do not display features of peripheral vasculopathy, nor do they present with circulating autoantibodies.

Lastly, but most intriguingly, the activation of IL-17A axis in our co-cultures was implicated in the modulation of fibroblast apoptosis. Apoptosis of SSc skin fibroblasts induced by co-cultured autologous PBMCs *in vitro* is likely to be influenced by different factors, one of which is the overexpression and activation of the death receptor Fas, as shown by our previous results (6). However, here we demonstrate that the high apoptotic rate of co-cultured fibroblasts added with rhIL-17A was counteracted by the simultaneous treatment with a neutralizing α-IL-17RA mAb, thus demonstrating that IL-17A axis plays a role regardless other possible mechanisms.

To date, little is known about IL-17A implication in programmed cell death. Arif et al. (44) reported that circulating IL-17+ cells autoreactive against several β-islet antigens are detectable in the blood of type I diabetes mellitus patients and induce β-cell death *via* activation of STAT1 and (NF)-kB transcription factors. Moreover, Su et al. (45) demonstrated that IL-17A is crucial in cardiomyocyte apoptosis through STAT3-iNOS pathway activation. On the contrary, other authors described an antiapoptotic activity played by IL-17A in other disease settings (46, 47). In particular, Kim et al. (47) reported that fibroblast-like synoviocytes exposed to IL-17 develop morphological and functional changes in mitochondrial proteins involved in autophagy resulting in resistance to apoptosis.

Our study has some limitations. First, all data come from *in vitro* experiments, as no other options are so far available to explore T cell–fibroblast interactions in SSc. Second, we did not use controls. However, other authors demonstrated that IL-17A stimulation has similar effects in SSc and healthy control fibroblasts (13, 19), thus suggesting that its pro/antifibrotic function is strictly depending on the microenvironment. Moreover, we demonstrated in previous studies (5, 6) that T cell–fibroblast interactions *in vitro* are specific to SSc. Taken together, our data and data from previous studies from independent groups (6, 7) suggest that T cell response in SSc might be also aimed to contrast aberrant TGF-β-dependent myofibroblast activation that is seen in this disease and that IL-17A might play a prominent dual role in this scenario (10–14), bridging a T cell response from a protective role to a profibrotic one in the long term. More studies are needed because of the paucity of data and inconsistent evidences from experimental models of fibrosis. Given so, the lack of clinical trials with IL-17 modulators in SSc is not surprising. Future lines of research are necessary, in particular to explore the proapoptotic potential of IL-17A to be translated in the clinical setting.

## Data Availability Statement

The datasets generated during and/or analyzed during the current study are available from the corresponding authors on reasonable request.

## Ethics Statement

The studies involving human participants were reviewed and approved by the Ethics Committee of the Department of Internal and Experimental Medicine F.Magrassi-A. Lanzara of the Second University of Naples. The patients/participants provided their written informed consent to participate in this study.

## Author Contributions

SV designed the study, planned and performed experiments, analyzed data, and drafted the manuscript. GB performed experiments, analyzed data, and critically revised the manuscript. BR, AB, GP, LP, and LV performed experiments and critically revised the manuscript. RD designed the study, planned experiments, analyzed data, and drafted the manuscript. All authors contributed to manuscript revision, read, and approved the submitted version.

### Conflict of Interest

SV received consultancy fees from Boehringer-Ingelheim and Thermo-Fischer, speaking fees and/or educational support from Abbvie, Roche, Pfizer, and BMS. However, no specific funding was received to carry out the work described in this manuscript. The remaining authors declare that the research was conducted in the absence of any commercial or financial relationships that could be construed as a potential conflict of interest.
