# Supplementary material for: T-Cell Proapoptotic and Antifibrotic Activity Against Autologous Skin Fibroblasts in vitro Is Associated With IL-17A Axis Upregulation in Systemic Sclerosis
Source: Front Immunol. 2020 Feb 27;11:220. doi: 10.3389/fimmu.2020.00220 (PMC7056890; doi:10.3389/fimmu.2020.00220)
Supplement: Supplementary file 1 [file Data_Sheet_1.doc]

**Supplementary table 1.Demographic, serological, and clinical features of SSc patients.**

| **Age** | **Sex** | **Subset*** | **Autoantibodies** | **Disease duration†** |
| --- | --- | --- | --- | --- |
| 37 | F | dcSSc | Anti-Scl-70 | 1 y |
| 23 | F | dcSSc | ANA | 0.6 y |
| 47 | F | dcSSc | ANA | 1.3 y |
| 30 | M | dcSSc | Anti-Scl-70 | 0.7 y |
| 63 | F | dcSSc | Anti-Scl-70 | 1.1 y |
| 48 | F | dcSSc | Anti-Scl-70 | 2.6 y |
| 32 | M | dcSSc | Anti-Scl-70 | 0.5 y |
| 47 | F | dcSSc | Anti-Scl-70 | 1 y |
| 27 | F | dcSSc | Anti-Scl-70 | 1.2 y |

SSc = systemic sclerosis; F = female; M = male; dcSSc= diffuse cutaneous systemic sclerosis; anti-Scl-70 = anti-Scl-70 antibodies; ANA = antinuclear antibodies; y = year

*Classified on the basis of skin involvement extent according to LeRoy and Medsger (20). †Disease duration is intended from the onset of Raynaud’s phenomenon, where early is a disease duration< 3 years.

**Supplementary table 2. Self-designed primers** for gene expression analysis.

| **Gene** | **Forward** | **Reverse** |
| --- | --- | --- |
| *IL6* | 5́ -CCCTGAGAAAGGAGACATGTAAC-3́ | 5́-CCTCTTTGCTGCTTTCACACATG-3́ |
| *IL17A* | 5´-CCTCATTGGTGTCACTGCTAC-3´ | 5´-GATTCCTGCCTTCACTATGG-3´ |
| *IL17RA* | 5´-ACCAGTTTTCCGCACATGGA-3´ | 5´-GCGCAGGTATGTGGTGCAT-3´ |
| *IL17RC* | 5́ -AGGGGCAAGAGCTGGGTCTGT-3́ | 5́-CCGGAGCAGGCACGATGTCC-3´ |
| *CXCL1* | 5´-AACCGAAGTCATAGCCACAC-3´ | 5´-CCTCCCTTCTGGTCAGTTG-3´ |
| *CCL2* | 5´-CTCGCTCAGCCAGATGCAATC-3´ | 5´-AAGTTATAACAGCAGGTGACT-3´ |
| *CCL3* | 5’-AGTTCTCTGCATCACTTGCTGC-3´ | 5´-GAATCTGCCGGGAGGTGTAG-3´ |
| *COL1A1* | 5´-TCAAGAGAAGCCTCACGATGG-3´ | 5´-TCACGGTCACGAACCACATT-3´ |
| *COL3A1* | 5´-GGCATGCCACAGGGATTCT-3´ | 5´-GCAGCCCCATAATTTGGTTTT-3´ |
| *CTGF* | 5´-TTGCGAAGCTGACCTGGAAGAGAA-3´ | 5´-AGCTCGGTATGTCTTCATGCTGGT-3´ |
| *TGFβR2* | 5´-CTGTGTCGAAAGCATGAAGG-3´ | 5´-GGTAGTGTTTAGGGAGCCGTC-3´ |
| *SMAD3* | 5´-GAACGTCAACACCAAGTGCAT-3´ | 5´-ACGCAGACCTCGTCCTTCT-3´ |

**Supplementary figure 1. *IL6* expression in dose-response experiments using hrIL-17A and α-IL-17RA mAb in human SSc skin fibroblasts.**

**
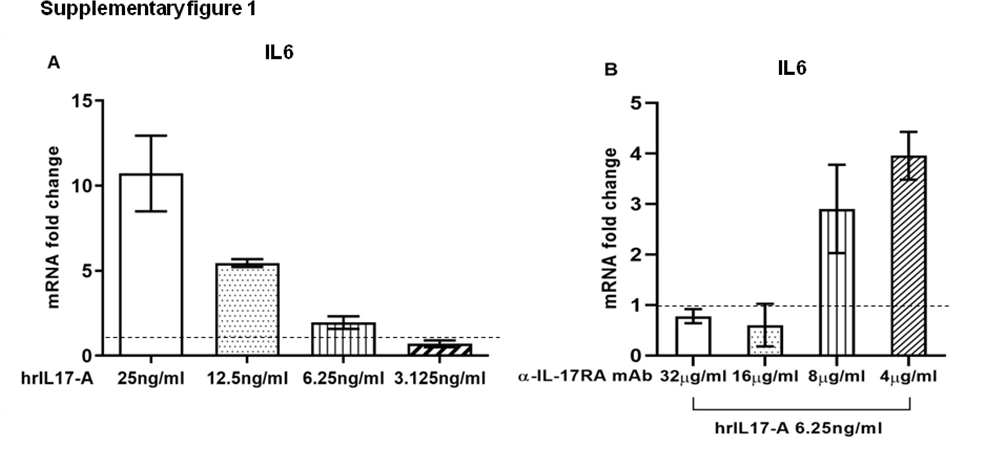
**

Results are fold changes in mRNA levels of *IL6* in Real-time PCR. Panel A shows the expression of *IL6*mRNA in SSc skin fibroblasts following 24 h stimulation with hrIL-17A at different concentrations, as indicated. Minimal effective concentration to obtain an increase in *IL6* mRNA levels of at least 1.5 fold was 6.25ng/ml.The dotted line indicate the expression of *IL6* mRNA in control cells treated with 0.1% BSA. The Panel (B) shows the expression of *IL6* mRNAin SSc skin fibroblasts following 24 h treatment with α-IL-17RA, added 1 h before hrIL-17A 6.26ng/ml, at different concentrations, as indicated.Minimal effective concentration to obtain a reduction of *IL6* mRNA levels to 1 fold or lower was 6.25ng/ml. The dotted line indicate the expression of *IL6* mRNA in control cells treated with goat isotype IgG at similar concentrations.

hrIL-17A = human recombinant IL-17A; α-IL-17RA = anti-IL-17 receptor A monoclonal antibody; SSc = systemic sclerosis

**Supplementary figure 2. Levels of *TGFB1*, *IL4* and *IL1B* in co-cultured PBMCs from early dcSSc patients.**


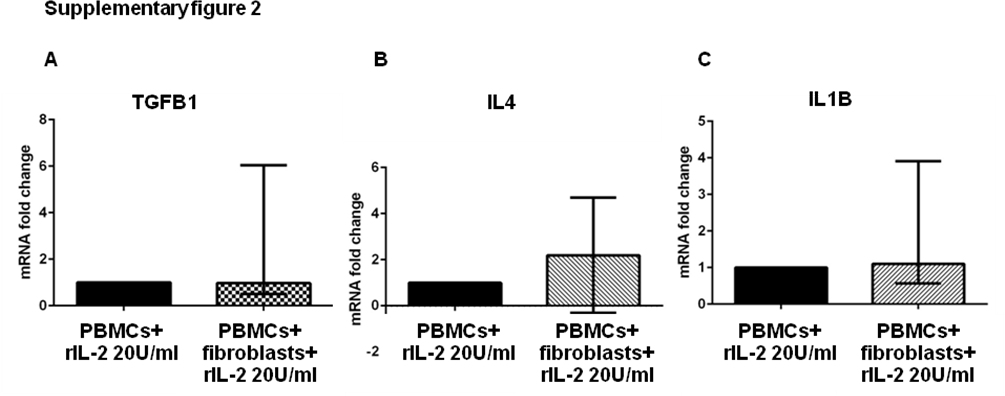


Results are fold changes in mRNA levels of target genes in Real-time PCR. Panel (A) shows the expression of *TGFB1* in PBMCs co-cultured with autologous skin fibroblasts and hrIL-2 20 U/ml as compared to PBMCs cultured in presence of hrIL-2 20U/ml only. Panel (B) shows the expression of *IL4* in PBMCs co-cultured with autologous skin fibroblasts and hrIL-2 20 U/ml as compared to PBMCs cultured in presence of hrIL-2 20U/ml only. Panel (C) shows the expression of *IL1B* in PBMCs co-cultured with autologous skin fibroblasts and hrIL-2 20 U/ml as compared to PBMCs cultured in presence of hrIL-2 20U/ml only. Data are median and IQR ranges, Wilcoxon signed rank test.

PBMCs = peripheral blood mononuclear cells; dcSSc = diffuse cutaneous systemic sclerosis; hrIL-2 = human recombinant IL-2; IQR = interquartile range
